# Impact of a multi-strain probiotic administration on peri-rectal colonization with drug-resistant Gram-negative bacteria in preterm neonates

**DOI:** 10.3389/fped.2022.1002762

**Published:** 2022-11-02

**Authors:** Marwyn Sowden, Evette van Niekerk, Andre Nyandwe Hamama Bulabula, Angela Dramowski, Andrew Whitelaw, Jos Twisk, Mirjam Maria van Weissenbruch

**Affiliations:** ^1^ Division of Human Nutrition, Department of Global Health, Faculty of Medicine and Health Sciences, Stellenbosch University, Cape Town, South Africa; ^2^ Infection Control Africa Network – ICAN, Cape Town, South Africa; ^3^ Department of Paediatrics and Child Health, Faculty of Medicine and Health Sciences, Stellenbosch University, Cape Town, South Africa; ^4^ Division of Medical Microbiology, University of Stellenbosch, Cape Town, South Africa; ^5^ National Health Laboratory Service, Tygerberg Hospital, Cape Town, South Africa; ^6^ Department of Epidemiology and Data Science, Amsterdam UMC, Amsterdam, Netherlands; ^7^ Department of Pediatrics, Amsterdam UMC, VU University Medical Center, Amsterdam, Netherlands

**Keywords:** multidrug-resistant Gram negative bacilli, neonate, probiotic, premature (babies), rectal swab

## Abstract

**Background:**

Infections caused by drug resistant Gram-negative bacteria (DR-GNB) are a major health concern for hospitalized preterm neonates, globally. The aim of this study was to investigate the effect of a multi-strain probiotic on the incidence of rectal colonization with DR-GNB in preterm neonates.

**Methods:**

A double-blind, placebo-controlled, randomized clinical trial was conducted including 200 neonates, randomly allocated to a multi-strain probiotic (*n* = 100) or placebo (*n* = 100).

**Results:**

Fifteen percent of the neonates showed peri-rectal colonization with DR-GNB on the day of enrolment indicating probable maternal-to-neonate (vertical) bacterial transmission or environmental acquisition at time of delivery, with no difference between groups. Acquisition of further DR-GNB colonization was rapid, with an increase from 15% on the day enrolment to 77% by day 7 and 83% by day 14 of life. By day 7 (corresponding to early gut colonization), neonates in the probiotic group were 57% less likely to have peri-rectal DR-GNB colonization [OR: 0.43 (0.20–0.95); *p* = 0.04] and by day 14 (corresponding to late gut colonization), neonates in the probiotic group were 93% less likely to have peri-rectal DR-GNB colonization [OR: 0.07 (0.02–0.23); *p* < 0.001].

**Conclusion:**

Hospitalized neonates showed substantial peri-rectal colonization with DR-GNB at enrolment and further rapid acquisition of DR-GNB in the first 2 weeks of life. The use of a multi-strain probiotic was effective in reducing early and late neonatal gut colonization with DR-GNB.

**Clinical Trial Registration:**

The trial was registered at the Pan African Clinical Trial Registry (PACTR202011513390736).

## Introduction

Globally infections caused by drug resistant Gram-negative bacteria (DR-GNB) have increased significantly and are a leading public health concern, especially in neonates (1, 2). Both early-onset and healthcare-associated infections (HAI) can be caused by antimicrobial resistant (AMR) bacteria (3, 4). In low-and-middle income countries (LMIC), most neonatal HAI-blood stream infection (BSI) episodes are caused by GNB, with high rates of infection-attributable mortality, morbidity, and increased healthcare costs (5, 6).

The most frequently cultured Gram-negative (GN) pathogens from neonatal BSI include *Klebsiella pneumoniae*, *Escherichia coli*, *Enterobacter* spp., *Serratia marcescens*, *Acinetobacter baumannii* and *Pseudomonas aeruginosa* (7–9). Factors contributing to neonatal colonization and/or infection with GN pathogens include poor compliance with hand hygiene, equipment and environmental cleaning recommendations, overcrowding, low nurse to patient ratios, overuse of broad spectrum antibiotics and delay in introducing breastmilk (10–12). A recent study in the neonatal wards at Tygerberg Hospital, South Africa showed that the highest aerobic colony counts were seen in moist surfaces, e.g., sinks, milk kitchen surfaces, humidifiers, and suction tubing. The organisms cultured were mainly *Enterococci*, *S. marcescens*, *K. pneumoniae*, *S. aureus* and *A. baumannii* (11). Due to their prematurity and absence of a suckling reflex, preterm neonates are mainly fed *via* a feeding tube. A review by Parker et al. indicated that feeding tubes can potentially contain pathogenic as well as antibiotic resistant bacteria (13).

A diverse community of organisms can be found in the gastrointestinal tract, referred to as the gut microbiome. Gut colonization is often delayed in preterm neonates, and these neonates also have limited number of species present (14). The most frequent species first seen in preterm neonates are *Enterobacteria* and *Streptococci* with a delayed colonization with *Bifidobacteria* (15). The lower the gestational age at birth, the slower the microbiome development and the lower the likelihood of *Bifidobacteria* predominance of the gut microbiome (16). Analysis of microflora in stool samples of extremely premature infants showed that the microflora of preterm infants changes progressively during the first 8 weeks of life, but that the diversity is exceptionally low. Concerning is the colonization with *Staphylococci* during the first 4 weeks of life, which poses an elevated risk for late onset sepsis (17). An increased risk of both necrotizing enterocolitis (NEC) and late-onset sepsis (LOS) have been observed in neonates with an altered microbiome (18). A predominance of aerobic cocci and reduced abundance of *Bifidobacteria* increases the risk of sepsis, while an increase in *Enterobacter* spp. and other *Proteobacteria* and reduction or absence of *Bifidobacteria* increases the risk of NEC and sepsis (16).

The large intestine can be a reservoir for many potential HAI pathogens including *Enterobacterales*, *Enterococci*, *Clostridioides difficile* and *Candida* species. Human-to-human spread of resistant bacteria can occur (19). Stephens et al. indicated that entire microbial communities and antibiotic-resistant bacteria can be spread *via* fomite transmission (20). Clinical infection may occur following intestinal acquisition of these pathogens, particularly in the presence of disruption to the gastrointestinal epithelial integrity and overgrowth of gut pathogen (19, 21). These conditions occur more frequently in preterm and low birth weight neonates, and in neonates with indwelling devices, prolonged hospital stay and prior antimicrobial use (21–23). In addition, most GN pathogens in LMIC exhibit AMR, making the treatment of HAI episodes difficult, e.g., extended beta-lactamase (ESBL) producing strains of *Klebsiella pneumonia* and *Escherichia coli* (24) and more recently emergence of carbapenem-resistant *Enterobacterales* (25).

A probiotic, as defined by the World Health Organization (WHO) is a live organism, which provides a benefit to the host when provided in adequate quantities (26). Probiotics can either be transient colonizers that alter the environment to allow for more permanent colonization by important commensal species or colonize the gut and modulate the gut microbiota (27, 28). They compete with other bacteria for nutrients and space, promote mucosal barrier function, inhibit mucosal pathogen adherence, and interact with the innate and adaptive immune system. Furthermore, probiotics may help to decolonize intestinal carriage of DR-GNB, through the production of antimicrobial substances, nitric oxide, and hydrolysis of pathogen receptors (29, 30). The ecosystem services framework can be applied to evaluate the neonatal gut microbiome. Through this system the goods and services obtained from the neonatal gut microbiome, can be categorized as supporting (capacity to colonize the gut), provisioning (ability of access and metabolize resources), or regulating (establish stable populations) (31).

In a systematic review and meta-analysis, Dermyshi et al. showed that probiotics (a single *Lactobacillus* species or a mixture of 2–3 bacterial species) reduced neonatal BSI rates by 12% and 19% respectively (32). Robertson et al. showed that a combination of *Lactobacillus* and *Bifidobacterium* supplementation led to a decreased incidence of both NEC and LOS (33). Although the modest benefits of probiotics for neonatal BSI prevention are promising, the optimal microbial strains, combinations, dosing, timing, and duration of supplementation, has not been definitively elucidated. Furthermore, it is not known whether probiotic supplementation in preterm neonates may modify the type, rate of acquisition, and antimicrobial-resistance patterns of perirectal colonizing GNB. We conducted a sub-study nested within a randomized controlled trial (RCT) to answer the question: does administration of a multi-strain probiotic reduce perirectal colonization with DR-GNB in preterm neonates?

## Methods

### Study design

A double-blind, placebo-controlled, randomized clinical trial was conducted.

### Study setting

The study was conducted in Tygerberg Hospital (TBH), Cape Town, South Africa. TBH has a capacity of 1,384 beds, with 132 neonatal beds including a 12-bed medical/surgical neonatal intensive care unit (NICU). Participants were recruited over a 6-month period (19 January to 27 June 2021).

### Study participants

Postnatally male and female preterm neonates, with a birth weight between 750 and 1,500 g and a gestational age <37 weeks were recruited, within 72 h of life. Neonates with major congenital malformations (including major gastro-intestinal abnormalities or surgery of the gastro-intestinal tract), birth asphyxia/neonatal encephalopathy, early onset sepsis [C-reactive protein (CRP) >10 mg/L in the first 72 h of life] and preterm neonates up for adoption, were excluded. Written parental informed consent was obtained.

### Sampling and randomization

Consecutive sampling was used: every preterm neonate meeting the inclusion criteria was selected for inclusion until the required sample size (200 neonates) was achieved. A pre-determined randomization sequence obtained from a statistician at Stellenbosch University was used to randomly allocate neonates to either the probiotic (intervention) group (*n* = 100) or placebo group (*n* = 100). The manufacturer of the probiotic packaged the products (probiotic and placebo) in identical fashion, except for a distinguishing pink and green sticker to facilitate allocation concealment. The randomization list indicated in series whether the infant should be allocated to the pink or green group. Every participant received their own product bottle, marked with their unique hospital sticker, in order to avoid cross-contamination and to ensure that the correct treatment was administered every day. The researcher, neonatal ward staff and laboratory staff were blinded to the allocation of probiotic versus placebo.

### Procedures

The probiotic used consists of *Lactobacillus acidophilus*, *Bifidobacterium bifidum* and *Bifidobacterium infantis* marketed as Labinic™ (Biofloratech, Surrey, United Kingdom). A standard dose of 0.2 ml was administered daily, providing approximately 2 billion colony forming units (CFUs) per day (approximately 0,67 × 10^9^ CFUs of each of the three organisms). The placebo consisted of medium chain triglyceride (MCT) oil and Aerosil 200, a stabilizer also used in Labinic.

Supplementation with the probiotic or placebo was initiated with the start of oral feeds. Supplementation was delayed if the neonate was nil per os (NPO) and discontinued if a neonate developed necrotizing enterocolitis (NEC) (Bells stage II or more). The probiotic/placebo was administered once daily in the neonate's feed (mother's own breast milk/donor breast milk/infant formula) before administration of the feed *via* an orogastric tube or if applicable, orally. Neonates were followed up from birth to a maximum of 28 days or death or discharge to peripheral hospitals or home, whichever came first. At this time point supplementation was discontinued.

Data collected at enrolment included estimated gestational age (early/late ultrasound or foot length), gender, birth weight, type of delivery and Apgar scores. Medical notes and laboratory data were reviewed daily, and data collected on type and volume of feeds received, anthropometric measurements, medication prescribed and when Kangaroo Mother Care (KMC) was initiated.

### Microbiological sampling and analysis

Peri-rectal swabs were collected at enrolment within 72 h of birth (day of enrolment), day 7 of life and day 14 of life reflecting baseline colonization status (probable maternally derived organism acquisition), early gut colonization and late gut colonization respectively. Lautenbach et al. indicated that peri-rectal swabs have a high sensitivity (90%) as well as specificity (100%) to correctly identify GNB (*E. coli* in their study) compared to stool samples (19). Since our study population was preterm infants, peri-rectal swabs would be the least invasive method to identify the DR-GNB. Sigma transwabs (MWE medical wire, United Kingdom) were used to perform peri-rectal swabs. The swab was pre-moistened in the Amies liquid transport medium of the swab tube and then swabbed twice around the peri-rectal area while rotating the swab tip. Swab tips were inoculated onto selective media (ChromID ESBL and ChromID CarbaSmart, BioMerieux, France) for isolation of ESBL- and carbapenem resistant GNB [including carbapenemase-producing Enterobacterales (CPE)], respectively. The plates were incubated aerobically for 18–24 h at 35°C and read the next morning. The presence of ESBL- and carbapenem-resistant organisms was indicated by the growth of pigmented colonies on the agar plate. The organism was then re-cultured on blood agar for 18–24 h at 35°C to ensure a pure colony. The organisms were then further identified to species level using API10E strips (BioMerieux, France). The manufacturer instructions were followed.

### Study outcomes

The primary outcome was acquisition and colonization by day 14 post-enrolment with ESBL- and/or carbapenem-resistant GNB (for the purposes of this study collectively referred to as drug resistant GNB (DR-GNB).

### Statistical analysis

The total sample size of the main study was 200, with 100 neonates per group (treatment and placebo groups). It was estimated using a published decrease (17%) in the proportion of perirectal colonization with drug-resistant bacteria (34). This sample size was estimated to detect a significant difference between the groups being compared (with Type I error at 0.05 and power at 80%). The total sample size required was allowing a 12% margin for study participant lost-to-follow-up. Continuous and categorical variables were compared using student *t* tests and Chi square tests respectively. A *p*-value below 0.05 was considered statistically significant. Logistic generalized estimating equations (GEE) were used to calculate the odds ratios for acquiring DR-GNB peri-rectal colonization by day 7 and day 14 post-enrolment. Both crude and adjusted analyses were performed. Adjustments were made for sepsis risk (infants with a CRP >10 mg/L and positive blood culture), HIV exposure, delivery mode, birthweight, gestational age, day of start of feeds, day of start of KMC and antibiotic use. Finally, we investigated whether the effect of probiotic supplementation on the presence of peri-rectal colonization with DR-GNB on average over time was different for infants with a different gestational age by adding the interaction between gestational age and product to the GEE analysis.

### Ethical approval

Ethical approval was granted by the Human Research Ethics Committee of the Faculty of Health Sciences of Stellenbosch University as well as Tygerberg Academic Hospital (S20/07/178). The trial was also registered at the Pan African Clinical Trial Registry (PACTR202011513390736).

### Role of the funding source

The funders of the trial had no role in the trial design, data collection, data analysis, data interpretation or writing of the report.

## Results

### Study participants

**Figure 1:**
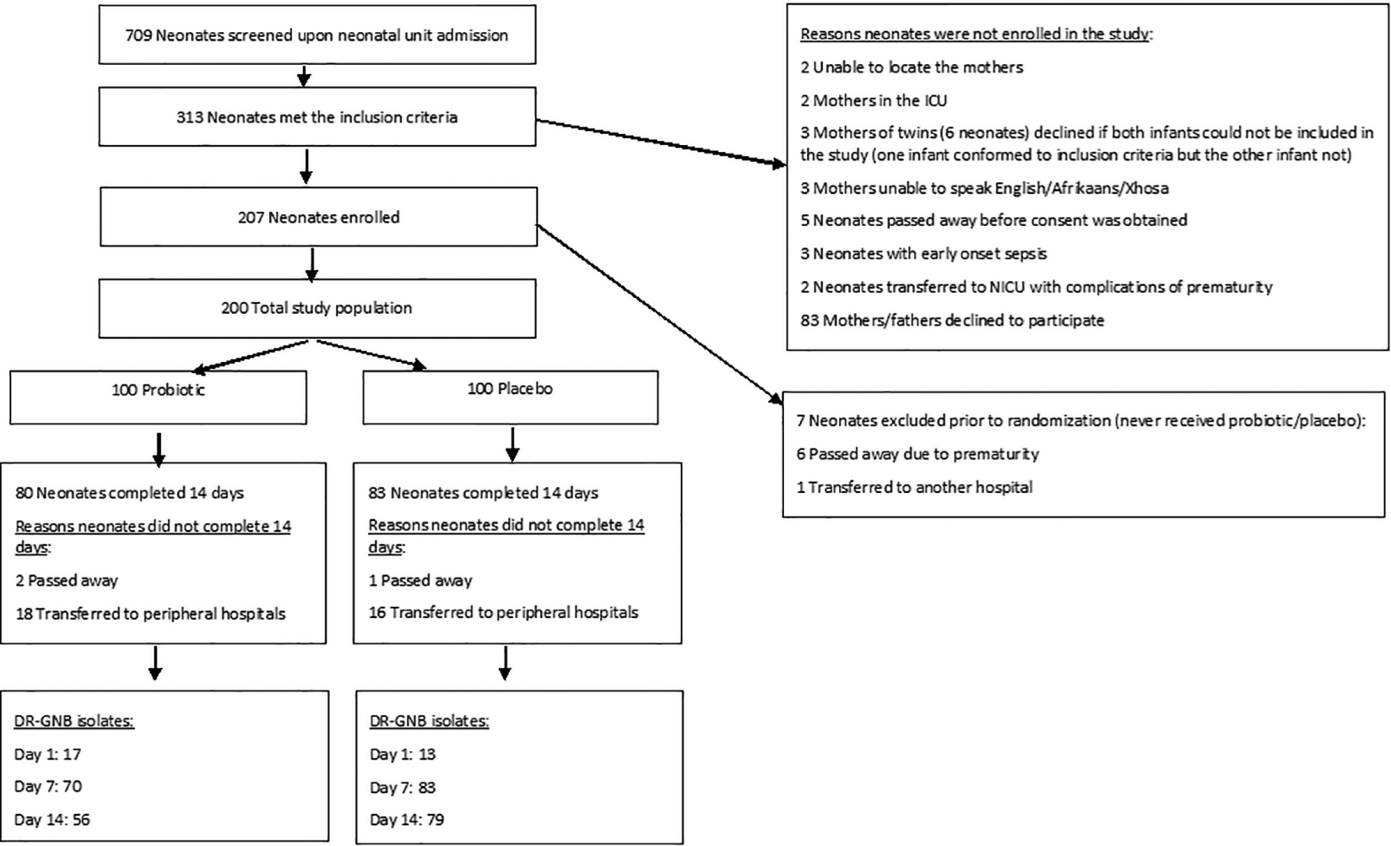
Flow diagram of the neonates included in the clinical trial, reasons for non-inclusion, randomization information as well as outcome. ICU, intensive care unit; NICU, neonatal intensive care unit; DR-GNB, drug-resistant Gram-negative bacilli.

A total of 709 neonates were screened for eligibility, after excluding the neonates that did not meet the inclusion criteria, 200 neonates were enrolled in the study (100 in each of the probiotic and placebo groups) (Figure 1). Of the 200 enrolled neonates, 163 (82%) completed the first 14 days of the study, 80 (80%) in the probiotic arm and 83 (83%) in the placebo arm.

### Demographics of the study participants

The demographic for the study participants were not significantly different as described in Table 1. The mean gestational age at birth between the two groups was similar—probiotic group 29 weeks; ±13.9 days (range 25–36 weeks) and the placebo group 30 weeks; ±13.5 days (range 25–34 weeks). The mean birthweight was also similar between the two groups—probiotic 1,174 g; ±226 g (range 780 –1,500 g) and placebo 1,150 g; ±230 g (range 750 –1,495 g). The majority of infants were born *via* caesarean section (*n* = 146; 73%). KMC was initiated on average at day 9.2 (range 3–21 days) in the probiotic group, and 9.1 (range 2–21 days) in the placebo group. Day of first feed was also similar between the probiotic and placebo group: 3.1 days; ±1.125 days (range 0–6 days) versus 3.0 days; ±0.985 days (range 2–6 days) respectively.

**Table 1:** Characteristics of the infants enrolled.

	Probiotic group (*n* = 100)	Placebo group (*n* = 100)
Mean gestational age at birth weeks ± days (range)	29; ±13.9 (25–36)	30; ±13.5 (25–34)
Mean birthweight grams (range)	1,174; ±226 (780–1,500)	1,150; ±230 (750–1,495)
Time to enrolment (median, IQR)	2 (1–2)	2 (2–2)
**Enrolment day:**		
Birth (Day 1 of life) (*n*, %)	30 (30)	23 (23)
Day 2 (*n*, %)	64 (64)	69 (69)
Day 3 (*n*, %)	6 (6)	8 (8)
Day of first feed days (range)	3.1; ±1.1 (0–6)	3.0; ±1.0 (2–6)
KMC initiated days (range)	9.2 (3–21)	9.1 (2–21)

### Microbiological sampling and analysis

The breakdown of the DR-GNB organisms cultured over the day enrolment, day 7 and day 14 of life are described in Table 2. At enrolment (<72 h after birth), 15% of neonates were already perirectally colonized with DR-GNB. There was no difference in colonization status between the probiotic and placebo arms at enrolment (17% versus 13% respectively; *p* = 0.553). By day 7, 71% of the probiotic group and 83% of the placebo group were colonized with DR-GNB (*p* = 0.060). The difference was more pronounced by day 14 with DR-GNB colonization present in 70% of the probiotic group and 95% of the placebo group (*p* < 0.001).

**Table 2:** Serial prevalence of rectal colonization with DR-GNB in preterm neonates.

	Total *n* (%)	Probiotic *n* (%)	Placebo *n* (%)	*p*-value
**Swab on day of enrolment**				
Total swabs collected	200	100	100	0.553
No growth	170 (85)	83 (83)	87 (87)	
DR-GNB present	30 (15)	17 (17)	13 (13)	
**Swab day 7 of life**				
Total swabs collected	199	99	100	0.060
No growth	46 (23)	29 (29)	17 (17)	
DR-GNB present	153 (77)	70 (71)	83 (83)	
**Swab day 14 of life**				
Total swabs collected	163	80	83	<0.001
No growth	28 (17)	24 (30)	4 (5)	
DR-GNB present	135 (83)	56 (70)	79 (95)	

**Figure 2:**
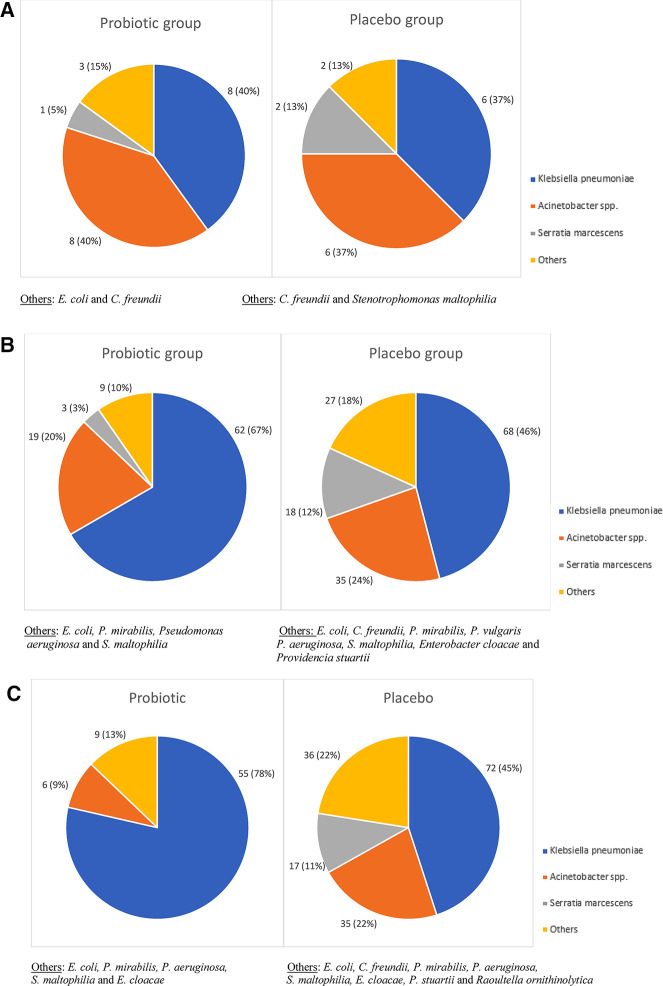
(**A–C**) Drug-resistant Gram-negative pathogens isolated from rectal swabs in preterm neonates by day of swab collection. (**A**) DR-GNB pathogens isolated from the day of enrolment swab. (**B**) DR-GNB pathogens isolated from the day 7 swab. (**C**) DR-GNB pathogens isolated from the day 14 swab.

The three most frequently cultured organisms included *Klebsiella pneumonia*e, *Acinetobacter* spp. and *Serratia marcescens*. The relative abundance of these is demonstrated in Figure 2. On the day of enrolment the most common DR-GNB isolated in both groups were *K. pneumoniae* (*n* = 8; 40% in the probiotic group and *n* = 6; 37.5% in the placebo group) and *Acinetobacter* spp. (*n* = 8; 40% in the probiotic group and *n* = 6; 37% in the placebo group), with lower *S. marcescent* numbers (*n* = 1; 5% in the probiotic group and *n* = 2; 13% in the placebo group). There was no difference in pathogen spectrum between study arms (*p* = 0.553).

At day 7 of life, *K. pneumoniae* (*n* = 62; 67% in the probiotic group and *n* = 68; 46% in the placebo group) and *Acinetobacter* spp. (*n* = 19; 20% in the probiotic group and *n* = 35; 24% in the placebo group) remained the most frequently isolated DR-GNB. There was a difference, although not statistically significant in the prevalence of peri-rectal carriage of DR-GNB between the two groups (*p* = 0.060). Of note, was a significant difference in the prevalence of *S. marcescens* carriage (*n* = 3; 3% in the probiotic group versus *n* = 18; 12% in the placebo groups; *p* = 0.032).

At day 14 of life, *K. pneumoniae* remained the dominant peri-rectal colonizing DR-GNB in the probiotic group (*n* = 55; 78%) and placebo group (*n* = 72; 45%), with *Acinetobacter* spp. in higher numbers in the placebo group (*n* = 35; 22%) than the probiotic group (*n* = 6; 9%). Of interest to note that there was no *S. marcescens* isolated cultured in the probiotic group, compared to *n* = 17; 11% in the placebo group. There was a significant difference in the overall DR-GNB carriage prevalence between the two groups (*p* < 0.001), as well as a difference in the frequency of *Acinetobacter* spp. and *S. marcescens* isolated from the two groups (*p* = 0.039 and *p* < 0.001 respectively) (Figure 2).

### Effects of probiotic supplementation

Infants supplemented with probiotics showed a significant reduction in the likelihood of peri-rectal colonization with DR-GNB at day 7 and 14 of life (Table 3). When adjusting for other variables (septic risk, HIV exposure, delivery mode, birthweight, gestational age, day of start of feeds, day of start of KMC and antibiotic use), the odds for isolating one/more DR-GNB on a peri-rectal swab at day 14 in the probiotic group was 0.24 as high as the odds for peri-rectal carriage of DR-GNB in the placebo group (95% CI: 0.12–0.47; *p* < 0.001).

**Table 3:** Crude and adjusted odds ratios for the effect of probiotic supplementation on the presence of peri-rectal colonization with DR-GNB over time.

Day of life	Crude	Adjusted
At day 7	0.51 (0.26–1.00); *p* = 0.05	0.43 (0.20–0.95); *p* = 0.04
At day 14	0.11 (0.03–0.33); *p* < 0.001	0.07 (0.02–0.23); *p* < 0.001
On average over time	0.30 (0.17–0.55); *p* < 0.001	0.24 (0.12–0.47); *p* < 0.001

The positive effects of probiotic supplementation appeared to be age dependent with more pronounced effects seen in preterm infants born < 32 weeks gestation (Table 4).

**Table 4:** Crude and adjusted odds ratios for the effect of probiotic supplementation on the presence of peri-rectal colonization with DR-GNB on average over time stratified by the infant's gestational age at birth.

Gestational age at birth	Crude	Adjusted
26–28 weeks (*n* = 64)	0.24 (0.08–0.68); *p* = 0.008	0.23 (0.08–0.69); *p* = 0.009
29–32 weeks (*n* = 122)	0.30 (0.14–0.66); *p* = 0.003	0.26 (0.12–0.56); *p* = 0.001
33–36 weeks (*n* = 14)	1.04 (0.13–8.12); *p* = 0.972	1.02 (0.12–8.39); *p* = 0.988

## Discussion

Antimicrobial resistance in Gram negative bacilli is of concern worldwide. Giuffre et al. conducted a 5-year prospective cohort surveillance study in Italy. They described an upward trend in the prevalence of intestinal colonization by DR-GNB (23). ESBL and carbapenem-resistant Enterobacteriaceae (CRE) both present a huge clinical problem worldwide as it causes a high morbidity and mortality rates (35). Literature describes that colonization with MDR-GNB is a risk factor for the development of BSI—around 50% of infants colonized with MDR-GNB, (especial ESBL-producing bacteria) can develop BSI (36, 37).

This double-blind RCT investigated the effect of a multi-strain probiotic on the prevalence of peri-rectal colonization with DR-GNB in preterm neonates receiving a multi-strain probiotic supplementation versus placebo. On the day of enrolment, fifteen percent of the neonates showed peri-rectal colonization with either ESBL-producing or carbapenem resistant GNB, indicating probable maternal-to-neonate (vertical) bacterial transmission or environmental acquisition at time of delivery, with no difference between groups. On day 7 (corresponding to early gut colonization), and day 14 (corresponding to late gut colonization) neonates in the probiotic group were less likely to have peri-rectal DR-GNB colonization compared to the placebo group.

The probiotic and placebo group started off with *K. pneumoniae* and *Acinetobacter* spp. as the two main DR-GNB organisms cultured. The pattern in DR-GNB organisms in the probiotic group changed over time showing *K. pneumoniae* dominance over *Acinetobacter* spp. In contrast the DR-GNB in the placebo group retained a higher proportion of *Acinetobacter* spp. and *S. marcescens* over time. A 10-year retrospective analysis of neonatal HA-BSI data from TBH showed that the following bacteria (*Klebsiella species*, *Staphylococcus aureus*, *S. marcescens*, *Enterococcus species* and *Acinetobacter baumannii*) were the pathogens most frequently cultured (38).

Previous studies found encouraging results for probiotic use to reduce carriage of pathogens in the neonatal gastrointestinal tract. One postulated mechanism of effect is that probiotic bacteria produce bacteriocins that enhance mucosal integrity and reduce pathogenic bacterial proliferation and resistance (39). Probiotic bifidobacterial strains can reduce AMR gene carriage by 90% compared to controls, e.g., Casaburi et al. showed that *Bifidobacterium infantis* can change the neonate gut microbiome and lower the abundance of common gut phyla such as *Proteobacteria* and *Firmicutes* (which include the genera *Escherichia* and *Clostridium*) (40). Nguyen et al. also found that the supplementation of *Bifidobacterium infantis* led to a decrease of *Klebsiella*, *Staphylococcaci* and *Enterobacteriaceae* in preterm infants. Further there was a high abundance of *Bifidoacteria* and most importantly a decreased in enteric inflammation (41). Roy et al. supplemented preterm neonates (<37 weeks, <2,500 g at birth) with *Bifidobacterium infantis*, *Lactobacillus*, and *Bifidobacterium lactis*. They showed that the supplemented group had a reduction in enteral fungal colonization, a reduction in fungal sepsis, earlier establishment of full enteral feeds and a reduced duration in hospital stay (30). Esaissaen et al. supplemented extremely preterm infants with *Bifidobacterium longum* and *Lactobacillus acidophilus*. They indicated that these infants had a high relative abundance of *Bifidobacterium* at 1 week of age, after receiving the supplement for only a few days, despite the high usage of antibiotics. Thus, the probiotic supplement induced colonization resistance and alleviated the harmful effects of antibiotic use on the gut microbiome (42). Van Best et al. studied the effect of probiotics on the developing microbiome. They concluded that probiotic supplementation reduced the abundance of organisms that are known to induce proinflammatory responses (43). Duar et al. used the ecosystem services framework to show that *Bifidobacterium longum* subsp. *infantis*, a gut symbiont, can effectively use human milk oligosaccharides (HMO) and change it into organic acids (mainly lactate and acetate). These organic acids alter the intestinal environment and prohibit the growth of pH-sensitive populations, known as enteric pathogens, of which many carry antibiotic resistant genes. Only *B. infantis* have the complete pathway necessary for intracellular HMO-transport and degradation (31).

A limitation of the study may be that culture-based methods using selective media were used to determine carriage status. While culture-based detection is likely less sensitive than molecular assays, the ChromID ESBL selective agar used in this study has been shown to have sensitivity of 88% (44), and the CarbaSmart a sensitivity of 90% (45). It is likely that a small proportion of children carrying MDR-GNB may have been missed using this approach, as opposed to a molecular screen. On the other hand, use of culture allows for identification of the resistant pathogens, as opposed to only detecting resistance genes in the sample. While culture will miss fastidious organisms, the medium we used supports the growth of common facultative Gram-negative pathogens very well. We did not characterize the resistance genes present in the cultured isolates which would have provided additional epidemiological information; this was a consequence of resource constraints. A maternal peri-rectal swab would have provided additional information regarding the maternal to neonate bacterial transfer, and future studies including both maternal and environmental swabs would be valuable in describing the relative contributions of maternal microflora and environmental flora to the carriage of resistance in neonates.

## Conclusion

Hospitalized neonates showed substantial peri-rectal colonization with DR-GNB at enrolment and further rapid acquisition of DR-GNB in the first 2 weeks of life. The use of a multi-strain probiotic was effective in reducing early and late neonatal gut colonization with DR-GNB.

## Data Availability

The raw data supporting the conclusions of this article will be made available by the authors, without undue reservation.
